# Endobronchial Infection and Bacterial Lymphadenitis by *Gemella morbillorum* Leading to Airway Perforation and a Bronchopleural Fistula

**DOI:** 10.1155/2024/8850287

**Published:** 2024-07-17

**Authors:** Kaitlin N. DePrez, John Ferguson

**Affiliations:** ^1^ College of Osteopathic Medicine Rocky Vista University, 8401 S Chambers Rd, Englewood, Colorado 80112, USA; ^2^ Department of Internal Medicine Rocky Mountain Pulmonary and Critical Care, 3555 Lutheran Pkwy, Suite 150, Wheat Ridge, Colorado 80033, USA

## Abstract

**Introduction:** Necrotizing bronchial infection with severe infectious lymphadenitis is infrequently encountered and most commonly ascribed to *Aspergillus*, *Histoplasma*, and *Mycobacterium* species. We present a unique cause of severe airway destruction with lymphadenitis and bronchopleural fistula formation by the bacterium *Gemella morbillorum*.

**Case:** A 24-year-old man presented with acute symptoms of vomiting, fever, and shoulder pain. A CT of the chest demonstrated a large subcarinal mass encasing the central bronchi. The workup for malignant, fungal, and granulomatous etiologies was unrevealing, while blood cultures identified *G. morbillorum*. Fiberoptic bronchoscopy revealed a perforation of the right middle lobar bronchus and the formation of a bronchopleural fistula, resulting in a large hydropneumothorax with empyema. Despite antibiotic therapy, surgical intervention to repair the fistula, and ventilatory support, the progression of the bronchopleural fistula led to fatal respiratory failure.

**Conclusion:** In cases of severe mediastinal adenopathy in a young patient, bacterial lymphadenitis should be considered in the differential diagnosis with lymphoma, germ cell tumor, granulomatosis with polyangiitis, sarcoidosis, histoplasmosis, and inflammatory myofibroblastic tumor.

## 1. Introduction

Infection with the commensal bacteria, *Gemella morbillorum*, is rare in humans and is an infrequent pathogen implicated in mediastinal lymphadenitis.

Mediastinal lymphadenopathy in a young adult is commonly ascribed to lymphoma, inflammatory myofibroblastic tumor, sarcoidosis, or infection by either *Mycobacterium tuberculosis* or *Histoplasma capsulatum* [1]. Central bronchial bacterial infection is uncommon but has been reported in pathogens such as *Actinomyces* spp., *Finegoldia magna*, *Corynebacterium* spp., *Rhodococcus equi*, and *Streptococcus pyogenes* [2]. Invasive bronchial infection caused by fungal organisms has been more commonly recognized than bacterial infection and is often caused by *Histoplasma*, *Aspergillus*, and *Mucorales* species [2].

We herein report a case of bacterial mediastinal lymphadenitis and bronchopleural fistula caused by the bacteria, *G. morbillorum*.

## 2. Clinical Case Description

A 24-year-old man presented with 4 days of vomiting, fever, and severe right shoulder discomfort radiating to the neck. He lacked any significant medical history to include causes for immunodeficiency, had no significant exposures, and had not traveled outside of the state of Colorado. On admission, vital signs consisted of a temperature of 37.2°C, a heart rate of 98 beats/min, a blood pressure of 128/68 mm Hg, and an oxygen saturation of 93% on room air. Laboratory investigations were notable for a white blood cell count of 16,200 cells per microliter, hemoglobin of 12.9 g/dL, and respiratory PCR positive for Severe Acute Respiratory Syndrome Coronavirus-2 (SARS-CoV-2, aka COVID-19).

Empiric antibiotic treatment for community-acquired pneumonia was initiated with intravenous ceftriaxone (1 g every 24 h) and azithromycin (500 mg every 24 h). A pulmonary consultation was obtained.

**Figure 1:**
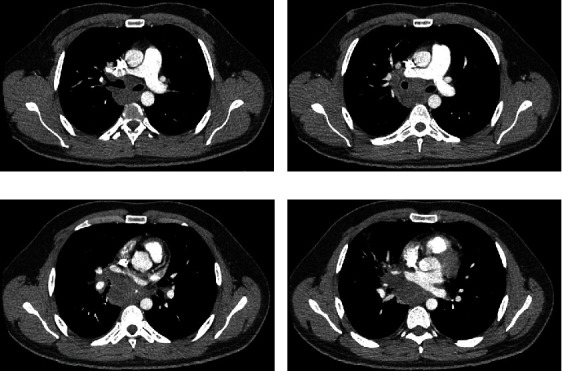
(a–d) CT scan of the chest demonstrating a large subcarinal mass encasing the right middle lobe bronchus. (c) Subtle perforation just distal to the right middle lobe orifice.

CT of the chest demonstrated a large subcarinal mass that encased both the right main bronchus and pulmonary artery, with associated airspace consolidation in the right middle lobe suggestive of bacterial pneumonia (Figures 1(a), 1(b), 1(c), and 1(d)).

Given the location and patient age, Hodgkin lymphoma, histoplasmosis, tuberculosis, and germ cell tumor were considered. Serum germ cell tumor markers alpha-fetoprotein and quantitative human chorionic gonadotropin were within normal limits. Antibodies to the human immunodeficiency virus, serum 1,3-beta-D-glucan, and serum *Aspergillus* galactomannan were not detected. Quantitative immunoglobulins and antinuclear antibodies were within normal limits, while antineutrophil cytoplasmic, myeloperoxidase, and serine protease antibodies were not detected. *Histoplasma* urine antigen was not detected, and *Histoplasma* mycelial antibody titer was less than 1:8, whereas *Histoplasma* yeast complement fixation (CF) antibody was elevated at 1:8. *Coccidioides* CF and *Blastomyces* antibodies were undetectable.

On hospital Day 2, the patient developed symptoms of sepsis with progressive fevers and hypotension and was transferred to the intensive care unit. Two thousand five hundred millimeters of Ringer's lactate was administered, leading to the restoration of perfusion. Blood cultures obtained on the day of admission demonstrated *G. morbillorum* in one of two bottles. Antibiotic sensitivities did not result, but antibiotics were empirically broadened to cefepime (2000 mg every 8 h) and vancomycin (1000 mg every 8 h).

**Figure 2:**
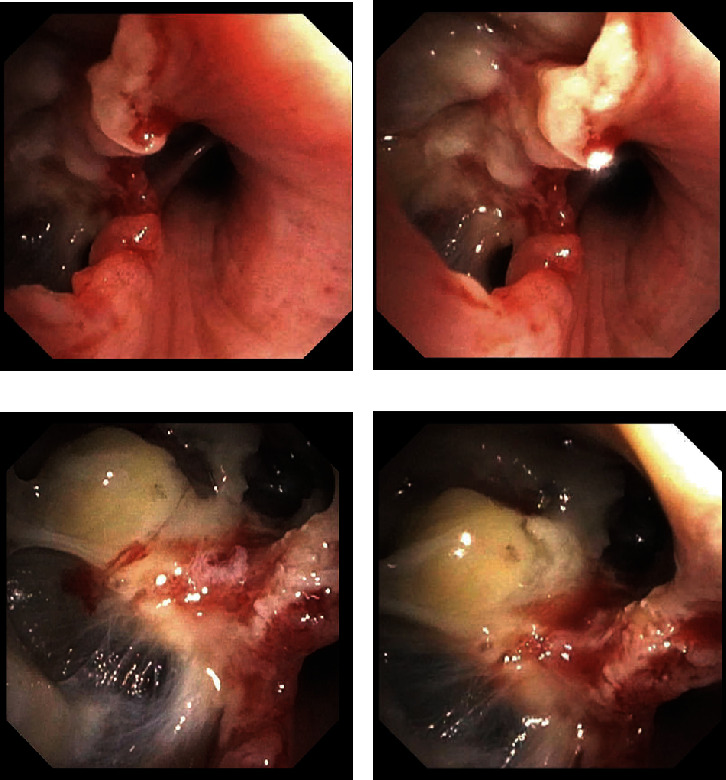
(a–d) Fiberoptic bronchoscopy of the right middle lobar bronchus revealing a bronchial erosion with ulcerated borders and mucinous material along a thin, fibrinous membrane–like structure. (a) Orifice of the right middle lobe with the lateral (RB4) and medial (RB5) airways visualized distally.

On hospital Day 5, fiberoptic bronchoscopy was performed, and fine needle aspiration of the subcarinal tissue yielded no immunoblastic or plasmablastic morphology, with no polymorphic lymphocyte population identified on flow cytometry. Bronchoalveolar lavage was performed in the right middle lobe fluid, with cultures negative for acid-fast bacilli, bacteria, and fungal organisms. *M. tuberculosis* polymerase chain reaction was negative. Airway inspection revealed erosive inflammation with near-perforation of the medial portion of the right middle lobar bronchus, approximately 1 cm distal to the bronchus origin, with ulcerated borders and mucinous material along a thin, fibrinous membrane–like structure (Figures 2(a), 2(b), 2(c), and 2(d)). A biopsy of the endobronchial erosion demonstrated acute organizing inflammation that stained negative for both acid-fast bacilli and fungal organisms.

**Figure 3:**
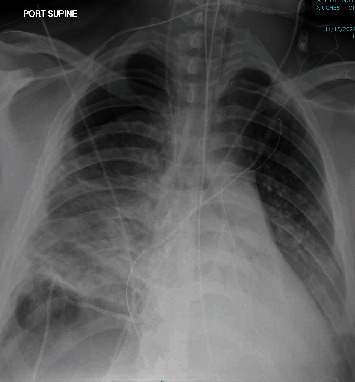
Chest X-ray after intubation, demonstrating a large right hydropneumothorax as a result of a bronchopleural fistula.

On hospital Day 6, following the progression of hypoxemic respiratory failure necessitating endotracheal intubation and subsequent exposure to positive pressure ventilation, a large hydropneumothorax developed (Figure 3). A tube thoracostomy was performed, revealing a high-volume bronchopleural fistula. Pleural fluid analysis was grossly characteristic of empyema, with pleural fluid glucose less than 1 mg/dL, lactate dehydrogenase 988 U/L, pH less than 6.8, and culture positive for *Prevotella nigrescens*.

**Figure 4:**
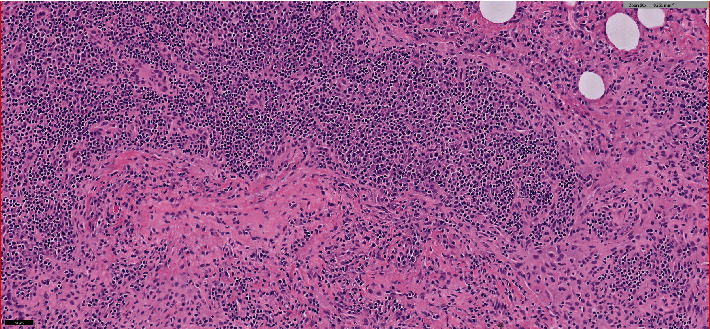
Histopathology of resected mediastinal mass revealing extensive infectious lymphadenitis.

Clinical markers of sepsis resolved, hemodynamics improved, the diffuse pulmonary infiltrates resolved, and surveillance blood cultures cleared; however, a considerable air leak persisted, which precluded liberation from invasive mechanical ventilation. Due to the location of the perforation within the right middle lobe bronchus, endobronchial stent placement was not feasible in consultation with interventional pulmonary. On hospital Day 10, a thoracotomy was performed to repair the continual bronchopleural fistula. After mobilization of the subcarinal tissue from the airway, a full-thickness perforation was visualized in the right middle lobe bronchus, adjacent to the bronchus intermedius, necessitating both right middle and right lower lobe lobectomy, as the location was not suitable for closure by either omentum or muscle flap. A locally disseminated, firm subcarinal lymph node was debulked thoroughly for pathologic evaluation. Histopathology of the resected tissue demonstrated extensive infectious lymphadenitis with no evidence of *Histoplasma* spp., *Mycobacterium*, germ cell tumor, myofibroblastic tumor, or lymphoma (Figure 4).

On hospital Day 15, while still requiring invasive mechanical ventilation, the patient developed progressive hypoxemia and hypercapnia with an inability to ventilate. A reoccurrence of a large-volume air leak was noted, indicating dehiscence of the repaired bronchial stump, resulting in severe hypoxemia and a fatal cardiac arrest.

## 3. Discussion

*G. morbillorum*, formerly *Streptococcus morbillorum*, is a facultative anaerobic, Gram-positive coccus that is part of the commensal flora of the oral and gastrointestinal tract. It is often misidentified as a viridans group *Streptococcus*, one that shares similar morphology and virulence to that of the *Gemella* species [3]. Human infection is rare, although it may be provoked by the use of intravenous drugs, excessive alcohol intake, diabetes mellitus, or poor dentition [4] [5]. Pneumonia with sepsis and bacteremia has been reported despite evident clinical symptoms of aspiration in both adults [6] and children [7].

Few cases have been reported in which *Gemella* spp. has led to infection of the mediastinum. Mediastinal lymphadenitis associated with *G. morbillorum* has been reported as a complication of endobronchial fine needle aspirate of the mediastinum with bacterial translocation [8]. Likewise, *Gemella* spp. mediastinitis has been reported to be related to odontic infection with the extension of a pharyngeal abscess [9, 10], bacterial translocation of a mycotic aortic aneurysm [4], blunt force trauma [5], and in association with esophageal adenocarcinoma [3].

Tuberculous lymphadenitis and histoplasmosis are more commonly implicated as causes of infectious mediastinitis as a result of compression of adjacent tissues and lymph node calcification [11]. A majority of cases of fibrous tissue infiltration of the mediastinum are linked to *Histoplasma capsulatum*, occurring in both active disease and the ensuing immunologic reaction [12]. Erosion of the bronchus from histoplasmosis or tuberculosis may occur when a caseous lymph node damages contiguous structures owing to a hyperreactive fibrous response. Bronchial erosion leads to obstruction of the airways with subsequent fistulization, including the formation of a bronchopleural fistula [13]. This process may develop over months and commonly produces calcification as a result of a robust fibrotic response. Despite the weakly elevated *Histoplasma* yeast CF, there was no evidence of mediastinal granulomatous inflammation or fungal elements in our patient.

Erosive central bronchial infection is uncommon but, when present, may lead to complications of bronchopleural fistula formation. Although largely limited to case reports, central airway infection with obstruction has been reported commonly in fungal diseases caused by both *Aspergillus* and *Mucorales* species [2]. These fungi frequently lead to endobronchial plaques and necrosis but are not reported to cause airway perforation, as was the case in our patient. *M. tuberculosis* is a well-established cause of bacterial endobronchial infection, and case reports have described similar endobronchial infections associated with *Actinomyces* spp., *Finegoldia* spp., *Rhodococcus equi*, and *Corynebacterium* spp. [2]. Likewise, except for those caused by endobronchial tuberculosis, bronchial perforation and bronchopleural fistula formation are rare. However, the formation of extensive bronchial fistulas has been reported as a result of fibrosing mediastinitis from a remote *Histoplasma* infection [14]. In our patient, *G. morbillorum* was identified as the primary pathogen that led to his bronchial perforation and was positive upon admission with features of sepsis. Although *Prevotella nigrescens* was identified in the empyema, this was a secondary pathogen caused by aspiration through the large bronchopleural fistula.

In the evaluation of extensive mediastinal lymphadenopathy, noninfectious causes should also be excluded, including inflammatory myofibroblastic tumor, germ cell tumor, lymphoma, granulomatosis with polyangiitis, and sarcoidosis. If transbronchial fine needle aspirate is nondiagnostic, surgical evaluation for these etiologies should be considered.

Repair of a bronchial perforation is indicated for patients with an early bronchopleural fistula and an acceptable surgical risk. Our patient had both the need for a definitive diagnosis as well as a large fistula that impeded adequate ventilation. Closure of the bronchopleural fistula is most commonly performed with a parietal pleura flap, pericardial fat pad, serratus anterior muscle flap, intercostal muscle flap, or gastric omentum flap to buttress the bronchus [15]. The pleural space should be thoroughly irrigated and debrided at the time of repair in the presence of an empyema [15]. Endobronchial treatment may be considered in patients with unacceptably high surgical risk; however, the proximity of the perforation immediately within the orifice of the right middle lobe precluded bronchial blocker placement such as a Watanabe Spigot or an Amplatzer occluder.

In our patient, airway perforation related to extensive subcarinal lymphadenitis led to the formation of an ultimately fatal bronchopleural fistula, which was unable to be successfully closed due to the severity of the infection, the fistula location, and the need for positive pressure ventilator support. Infectious mediastinal lymphadenitis not caused by *Aspergillus*, *Mycobacterium*, or *Histoplasma* species should be considered in patients with airway perforation after the exclusion of inflammatory or malignant causes.

## 4. Conclusion

• In cases of extensive mediastinal lymphadenopathy, careful airway inspection may demonstrate airway involvement and fistula formation
• Infectious mediastinal lymphadenitis may mimic lymphoma, germ cell tumor, inflammatory myofibroblastic tumor, and sarcoidosis
• Extensive subcarinal lymphadenopathy is a risk factor for the formation of a bronchopleural fistula

## Data Availability

All underlying data can be found in the manuscript.
